# The Increased Risk for Postinfluenza Pneumonia Among Cystic Fibrosis Carriers—A Population-Based Study

**DOI:** 10.1093/ofid/ofaf642

**Published:** 2025-10-23

**Authors:** Aaron C Miller, D Erik Boonstra, Joe E Cavanaugh, Mahmoud H Abou Alaiwa, Alejandro P Comellas, Douglas B Hornick, David A Stoltz, Philip M Polgreen

**Affiliations:** Department of Internal Medicine, Division of Infectious Diseases, University of Iowa, Iowa City, Iowa, USA; Department of Biostatistics, University of Iowa, Iowa City, Iowa, USA; Department of Biostatistics, University of Iowa, Iowa City, Iowa, USA; Department of Internal Medicine, Division of Pulmonary, Critical Care, and Occupational Medicine, University of Iowa, Iowa City, Iowa, USA; Department of Internal Medicine, Division of Pulmonary, Critical Care, and Occupational Medicine, University of Iowa, Iowa City, Iowa, USA; Department of Internal Medicine, Division of Pulmonary, Critical Care, and Occupational Medicine, University of Iowa, Iowa City, Iowa, USA; Department of Internal Medicine, Division of Pulmonary, Critical Care, and Occupational Medicine, University of Iowa, Iowa City, Iowa, USA; Department of Internal Medicine, Division of Infectious Diseases, University of Iowa, Iowa City, Iowa, USA

**Keywords:** cystic fibrosis carrier, influenza, pneumonia

## Abstract

**Background:**

Influenza is strongly associated with an increased risk for subsequent bacterial pneumonia. Moreover, cystic fibrosis (CF) carriers are at increased risk for some pulmonary infections. The purpose of this study was to determine whether CF carriers are at greater risk for postinfluenza pneumonia than noncarriers.

**Methods:**

Using MarketScan insurance claims data (2001–2023), we identified a study cohort of 38 047 CF carriers and a cohort of 380 470 matched controls. We conducted 2 analyses using these cohorts. First, we assessed individual-level risk for experiencing pneumonia following an influenza infection. Second, because many cases of influenza often do not result in medical visits, we conducted a cohort-level analysis comparing the weekly incidence of pneumonia between CF carriers and noncarriers while accounting for weekly Centers for Disease Control and Prevention–reported influenzalike illnesses across multiple influenza seasons.

**Results:**

At an individual level, we found that the odds of developing pneumonia following a diagnosis of influenza were approximately 34% greater among CF carriers compared with noncarriers. Second, we found that while the incidence of influenza is not elevated among CF carriers, the incidence rate of pneumonia was about 55% greater among CF carriers compared with our control population.

**Conclusions:**

Because 2%–11% of the population acquires influenza each year, and because there are >10–15 million CF carriers in the United States alone, a substantial number of cases of secondary pneumonia may be attributable to the CF carrier state.

## Teaser

Influenza is strongly associated with an increased risk for subsequent bacterial pneumonia. We found that the odds of developing pneumonia following a diagnosis of influenza were approximately 38% greater among CF carriers compared with noncarriers.

Cystic fibrosis (CF) is an autosomal recessive disease caused by deleterious mutations in the CF transmembrane conductance regulator (*CFTR*) gene, which encodes a chloride and bicarbonate channel [1]. The disease affects multiple organ systems, but almost all people with CF experience recurrent pulmonary infections [1]. These infections cause pulmonary exacerbations that contribute to bronchiectasis, diminished respiratory function [2], and death [3]. Pulmonary infections among people with CF typically involve bacterial pathogens, including *Staphylococcus aureus, Pseudomonas aeruginosa*, *Stenotrophomonas maltophilia*, and *Burkholderia cepacia* complex [4, 5]. Drug-resistant nontuberculous mycobacterial infections are also common in people with CF [6].

Viral pathogens can also be associated with pulmonary exacerbations of CF [7]. Indeed, pulmonary exacerbations among people with CF occur more frequently during the winter months, corresponding to the respiratory viral season [5]. In addition, pulmonary exacerbations associated with viral infections can also lead to worsening of respiratory function [8]. Collectively, these observations suggest that respiratory viruses adversely affect clinical outcomes in people with CF.

Multiple respiratory viruses have been associated with pulmonary exacerbations of CF [9], but influenza appears to be highly associated with CF exacerbations in both adults and children [7]. For example, children with CF are >10 times more likely to be hospitalized with influenza than children without CF or other chronic lung diseases [10]. The increased risk for pulmonary exacerbations associated with influenza is not surprising, given that influenza infections can alter pulmonary host defense though multiple mechanisms in people without CF [11], and the association of influenza with secondary bacterial infections (eg, bacterial pneumonia) in people without CF has been well described [12].

If people with CF are at increased risk for pulmonary exacerbations following an influenza infection, CF carriers, with just 1 *CFTR* gene mutation, may also be at increased risk for subsequent pulmonary infections following an influenza infection. Previously, it was thought that CF carriers were not at increased risk for CF-associated diseases [13, 14]. However, it is now clear that CF carriers are at increased risk for many diseases associated with CF, including respiratory infections [15] and bronchiectasis [16]. Accordingly, CF carriers may be at additional risk for secondary bacterial pneumonia after an influenza infection, compared with the general population.

The objective of this study was to evaluate the link between the CF carrier state and the risk for postinfluenza pneumonia. We conducted 2 analyses using a cohort of CF carriers and matched controls. First, we estimated the individual-level risk for experiencing pneumonia following an influenza infection. Second, because many cases of influenza do not result in medical visits [17], we conducted a secondary analysis at a cohort level, analyzing the weekly incidence of pneumonia across multiple influenza seasons.

## METHODS

### Data Source

We used data from the Merative MarketScan Research Databases [18], which include the Commercial Claims and Encounters and Medicare databases from 2001–2023 and the Multi-State Medicaid Database from 2014–2021. This data source is one of the largest databases of deidentified insurance claims in the United States and represents >256 million enrollees over the study period. These data include insurance claims from inpatient, outpatient, and emergency department visits, along with outpatient prescription medications and enrollment information. Enrollment information includes an enrolled family identifier and employee relation variable that can be used to link records between individuals enrolled in a family health insurance plan.

### Study Design and Population

For both analyses, we started with data from a matched-cohort study design that included a cohort of identified CF carriers and a matched control cohort of noncarriers. We first identified all individuals diagnosed as a CF carrier via genetic testing (*International Classification of Diseases, Ninth Revision, Clinical Modification* code V83.81 or *International Classification of Diseases, Tenth Revision, Clinical Modification* code Z14.1). We eliminated from inclusion any CF carrier who also had a diagnosis code for CF (see Supplementary Table 1). Next, we identified potential control patients from all enrollees without evidence of *CFTR* mutations using 2 criteria: (1) the individual had no diagnosis of CF carrier or CF and (2) none of the individual's enrolled family members were diagnosed with CF or as a CF carrier. From these enrollees without *CFTR*-related diagnosis codes, we identified a control cohort that was matched 10:1 to each CF carrier. Controls were randomly matched for exact sex, age, number of months of enrollment, and year of first enrollment. A 10:1 match was selected to maximize the size of the control population while minimizing the number of CF carriers excluded due to lack of matches. Note that if we were unable to identify 10 exact matches for each carrier, we allowed selected controls to have a slightly longer period of enrollment. An earlier version of this study cohort has been used previously to study a range of CFTR-related conditions, and a more extensive description of the study population and evaluation of potential biases has been described elsewhere (see Miller et al [15]).

### Individual Risk Analysis for Postinfluenza Pneumonia

For our primary analysis, we conducted a nested-case study of CF carriers and controls who experienced an influenza event. We estimated the odds of experiencing pneumonia following an influenza infection among CF carriers and matched controls. For both cohorts, we identified all distinct influenza diagnoses using the diagnosis codes provided in Supplementary Table 1. Influenza events were identified by the first influenza diagnosis that occurred ≥180 days after a previous influenza diagnosis. For each influenza event, we then determined whether the patient was diagnosed with pneumonia within 0–14 days of the initial influenza diagnosis for that event using the diagnosis codes in Supplementary Table 1. We chose 0–14 days because illness attributable to influenza can last up to 2 weeks [19]. We included cases where the patient was diagnosed with pneumonia and influenza concurrently. We also noted whether the pneumonia or influenza diagnosis occurred during a hospitalization.

To estimate the odds of experiencing pneumonia following an influenza infection, we used a logistic regression model in which the outcome designated whether the patient experienced pneumonia within 14 days of the given influenza event. For explanatory variables, we included an indicator for CF carrier status. Because the nested-study design breaks the exact match between CF carriers and controls, we also controlled for patient age group and sex, along with indicators for the year and month of the influenza event. Finally, we controlled for comorbid conditions using the 30 Elixhauser comorbidity indicators [20]; each indicator was constructed using all diagnoses a patient received on or within the 30 days before the index influenza event. As a secondary analysis we also evaluated the risk for being hospitalized with pneumonia, by restricting the 14-day pneumonia outcome to cases in which the pneumonia diagnosis was recorded during an inpatient stay.

### Analysis of Weekly-Level Pneumonia Incidence

Because many cases of influenza are not medically attended, the number of influenza events and pneumonia cases linked to influenza, are both underidentified. Thus, we formulated and fit a model of weekly pneumonia incidence as a function of CF carrier status and exogenous influenza activity. This model included all CF carriers and matched controls. We used Centers for Disease Control and Prevention (CDC)–reported influenzalike illness (ILI) incidence as an exogenous measure of influenza activity to account for community-level influenza activity.

For this analysis, we computed the number of pneumonia events that occurred each week for both CF carriers and matched controls. To model pneumonia incidence, we used a generalized linear model with a Poisson distribution and log link. The outcome of the model was the number of pneumonia cases in each week. To evaluate the risk among CF carriers, we included an indicator for CF carrier status. We also included a 1-week lag in the CDC ILI measure, to account for the timing between the initial influenza infection and secondary pneumonia. The model also included indicators for the year and month of observation, along with an offset term for the number of enrollee-days that were observed in the given week. Because incidence was computed using the entire matched cohort, which was matched on age, sex, and enrollment period, age and sex were not included as covariates in this model.

As a secondary analysis we evaluated whether CF carrier status conveyed an interactive effect with influenza incidence. Specifically, we evaluated whether CF carriers were at consistently greater risk for pneumonia than noncarriers or whether the level of CF carrier risk relative to controls increased or decreased with the underlying level of influenza incidence. To evaluate this effect, we compared models that included different combinations of indicators for CF carrier status, weekly ILI activity, and an interaction term between the two.

### Sensitivity Analysis

We conducted 2 sensitivity analyses. First, pneumonia typically occurs within 1 week of an influenza infection, so our primary analysis used a 1-week lag. However, we also evaluated models with no lag and with 2-, 3-, and 4-week lags in the ILI measure. In addition, we evaluated an internally computed measure of influenza incidence, defined as the number of cases of influenza each week in the entire MarketScan cohort divided by the number of enrollees that week. We then compared model performance (measured by the Akaike information criterion) and the impact on the effect of CF carrier status.

Second, we evaluated the sensitivity of our results to the inclusion or exclusion of periods of atypically high or low influenza incidence. Specifically, before 2009, our study cohort was smaller, and outcomes of pneumonia were sparser. The coronavirus disease 2019 (COVID-19) pandemic resulted in a substantial decline in influenza incidence after 2019. Thus, we reevaluated our results after the exclusion of data before 2009 and/or after 2019.

## RESULTS

We identified a study cohort of 38 047 CF carriers and a cohort of 380 470 matched controls. Table 1 summarizes characteristics of this study population. Most CF carriers are identified via genetic screening during preconception care. In addition, some CF carriers are identified via newborn screening. Thus, most enrollees were aged 18–29 or 0–4 years. In the overall cohorts, a slightly greater proportion of CF carriers had an influenza (8.5%) or pneumonia (5.0%) diagnosis, compared with controls (8.0% and 4.1%, respectively). CF carriers were also slightly more likely than controls to be hospitalized for pneumonia (1.1% vs 0.8%, respectively). Supplementary Figure 1 depicts the number of enrollees represented in the study cohorts across time.

**Table 1.** Study Population Characteristics

Characteristic	Patients, No. (%)^a^	
	CF Carriers (n = 39 833)	Matched Controls (n = 398 320)
Female sex	32 446 (85.3)	324 460 (85.3)
Age at first enrollment, years		
0–4	4119 (10.34)	41 190 (10.34)
5–17	2668 (6.7)	26 670 (6.7)
18–29	12 938 (32.48)	129 380 (32.48)
30–39	1291 (3.24)	12 910 (3.24)
40–49	366 (0.92)	3660 (0.92)
50–65	53 (0.13)	530 (0.13)
≥65	4119 (10.34)	41 190 (10.34)
Age at last enrollment		
0–4	2967 (7.45)	29 589 (7.43)
5–17	1558 (3.91)	16 111 (4.04)
18–29	12 047 (30.24)	120 989 (30.37)
30–39	17 450 (43.81)	172 964 (43.42)
40–49	4810 (12.08)	48 495 (12.17)
50–65	820 (2.06)	8379 (2.1)
≥65	181 (0.45)	1793 (0.45)
Enrollment duration, days		
Mean (SD)	1694.90 (1323.59)	1698.20 (1329.23)
Median (range)	1400 (1–8400)	1400 (2–8400)
Multi-State Medicaid Database	9894 (24.8)	98 940 (24.8)
Influenza		
Influenza ever diagnosed	3403 (8.54)	31 944 (8.02)
Total healthcare visit days with diagnosis, no.^b^	4350	40 469
Pneumonia		
Pneumonia ever diagnosed	2004 (5.03)	16 382 (4.11)
Hospitalized	455 (1.14)	3306 (0.83)
Total healthcare visit days with diagnosis, no.^b^	5579	33 206
Total hospitalizations, no.	736	4784
Concurrent influenza and pneumonia		
Any concurrent diagnosis	105 (0.26)	852 (0.21)
Total concurrent visits, no.	135	1185

Abbreviation: CF, cystic fibrosis.

^a^Data represent no. (%) of patients unless otherwise specified.

^b^Treatment for a given influenza or pneumonia event may involve healthcare interactions across multiple days.

**Figure 1.**
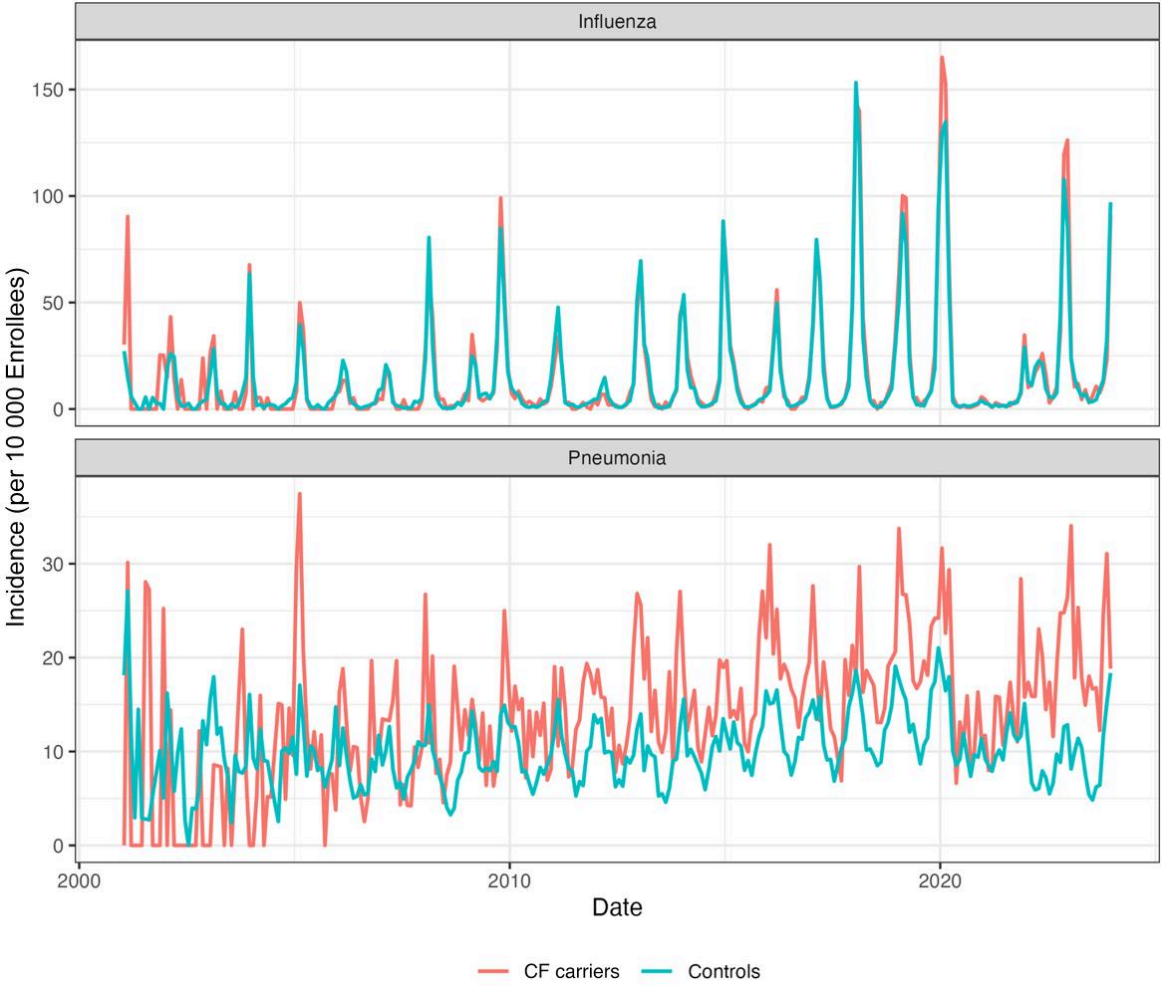
Monthly influenza and pneumonia incidence (per 10 000 enrollees) among cystic fibrosis (CF) carrier and matched control cohorts. There is little to no difference in monthly influenza incidence between CF carriers and controls. However, CF carriers tended to have a consistently higher incidence of pneumonia. The incidence for CF carriers before 2009 is less stable because of scarcity of cases of influenza and pneumonia; this is the period when the study cohort is less populated due to a lower frequency of genetic testing (see Supplementary Figure 1 for a summary of the size of the study population across time).

Figure 1 also displays the monthly incidence of pneumonia and influenza for CF carriers and controls across the study period. Supplementary Figure 2 depicts the incidence of influenza and pneumonia by age; this figure shows a bimodal distribution with risk for both influenza and pneumonia increase in the youngest and older age groups. From both these figures there appears to be little difference in the incidence of influenza between CF carriers and matched controls. However, CF carriers tend to have a consistently greater incidence of pneumonia across time and age groups than controls.

### Individual Risk Analysis

Supplementary Table 2 presents cohort characteristics of the influenza events included in the individual risk analysis. In total we identified 3820 index influenza events among CF carriers, of which 116 (3.0%) resulted in secondary pneumonia within 14 days. Comparatively, we identified 36 247 index influenza events among controls, of which 844 (2.33%) resulted in pneumonia. The unadjusted odds ratio for secondary pneumonia following influenza for CF carriers versus controls was 1.31. Table 2 presents the results of the logistic regression analysis estimating the odds for pneumonia following influenza. After accounting for patient age, sex, and time of infection, the odds ratio for secondary pneumonia was 1.34 (95% confidence interval [CI], 1.09–1.63) for CF carriers versus controls. Estimates for other covariates are presented in Supplementary Table 1.

**Table 2.** Regression Results for Study 1: Risk for Pneumonia Within 14 Days After an Influenza Event

Covariate^a^	OR (95% CI)	
	Any Secondary Pneumonia	Hospitalization for Secondary Pneumonia
CF carrier	1.34 (1.09–1.63)	1.29 (.75–2.20)
Age group, years		
0–4	1.38 (1.08–1.75)	1.24 (.65–2.35)
5–17	1.06 (.83–1.34)	0.85 (.44–1.66)
18–29	Reference	Reference
30–39	1.04 (.88–1.23)	0.94 (.62–1.45)
40–49	1.35 (1.06–1.72)	0.90 (.46–1.77)
50–64	1.98 (1.31–2.98 )	2.20 (.92–5.27)
≥65	4.10 (1.63–10.34)	5.29 (.98–28.64)
Female sex	0.99 (.82–1.19)	0.92 (.55–1.52)

Abbreviations: CF, cystic fibrosis; CI, confidence interval; OR, odds ratio.

^a^Models also included indicators for year and month (see Supplementary Table 1 for remaining effect estimates).

We also estimated the odds of being hospitalized for secondary pneumonia by restricting the outcome of pneumonia to cases that were diagnosed within a hospital stay. Among CF carriers with influenza, we identified 16 cases (0.42%) in which secondary pneumonia occurred during a hospital stay (see Supplementary Table 2). Among controls we identified 129 such cases (0.36%). Table 2 also presents the results of the regression analysis for pneumonia hospitalizations. After accounting for patient age, sex, and time of infection, the odds ratio for being hospitalized with secondary pneumonia was not statistically significant (1.29 [95% CI, .75–2.20]) for CF carriers versus controls.

### Analysis of Weekly-Level Pneumonia Incidence

For our final analysis, we modeled the weekly incidence of pneumonia as a function of CF carrier status, lagged exogenous influenza activity, month, and year. Supplementary Figure 3 depicts the weekly incidence of pneumonia for CF carriers and matched controls. In general, the incidence rate of pneumonia was higher for CF carriers than for matched controls for nearly all time points. However, this pattern is slightly harder to detect before 2009, when genetic screening was less common and fewer CF carriers were identified.

Table 3 reports results from our Poisson regression analysis, where we modeled the weekly incidence of pneumonia as a function of CF carrier status, lagged exogenous influenza activity, month, and year. After controlling for community ILI levels, month, and year, the incidence rate ratio (IRR) for pneumonia among CF carriers versus controls was 1.55 (95% CI, 1.50–1.60). As expected, lagged ILI was also positively associated with pneumonia incidence with a rate ratio of 1.09 (95% CI, 1.08–1.10) for each additional percentage point of weighted ILI.

**Table 3.** Regression Results for Study 2: Weekly Incidence of Pneumonia

	IRR (95% CI)
CF carrier	1.55 (1.50–1.60)
CDC ILI (with 1-wk lag)	1.09 (1.08–1.10)
Month	
January	Reference
February	0.97 (.92–1.02)
March	0.93 (.88–.97)
April	0.86 (.82–.91)
May	0.79 (.74–.83)
June	0.72 (.68–.77)
July	0.68 (.64–.72)
August	0.74 (.70–.79)
September	0.82 (.77–.87)
October	0.93 (.88–.98)
November	1.02 (.97–1.07)
December	1.05 (1.00–1.10)
Year	
2001	Reference
2002	0.83 (.58–1.17)
2003	1.12 (.82–1.53)
2004	1.00 (.74–1.36)
2005	1.15 (.86–1.55)
2006	0.90 (.67–1.21)
2007	1.00 (.75–1.34)
2008	0.91 (.69–1.22)
2009	1.07 (.80–1.42)
2010	1.02 (.77–1.36)
2011	1.15 (.87–1.53)
2012	1.12 (.84–1.49)
2013	1.05 (.79–1.40)
2014	1.13 (.85–1.50)
2015	1.23 (.93–1.63)
2016	1.40 (1.05–1.85)
2017	1.23 (.93–1.64)
2018	1.33 (1.00–1.77)
2019	1.49 (1.12–1.98)
2020	1.20 (.91–1.60)
2021	1.21 (.91–1.61)
2022	0.89 (.67–1.18)
2023	1.12 (.84–1.49)

Abbreviations: CDC, Centers for Disease Control and Prevention; CF, cystic fibrosis; CI, confidence interval; ILI, influenzalike; IRR, incidence rate ratio.

We conducted a secondary analysis to determine whether the effect of CF carrier status was additive or had an interactive relationship with underlying ILI activity. We found that the effect of CF carrier status is additive and that the risk of secondary pneumonia for CF carriers compared with controls does not deviate with the underlying level of influenza activity (see Supplementary Table 4 for details).

As a sensitivity analysis, we evaluated the number of lags for exogenous ILI to include in the model. Supplementary Table 5 presents these results in terms of both model performance (as assessed by Akaike information criterion and the Bayesian information criterion) and the primary effect estimate. Based on these results, the inclusion of a single 1-week lag term was deemed the most preferred model (see summary in Supplementary Table 5). Moreover, the primary effect estimate for CF carrier status was unchanged across model specifications.

Supplementary Table 6 reports the results of our second sensitivity analysis. We found that none of our estimates were affected by including or excluding data before 2009 or after 2019. All IRR estimates were statistically significant, and IRR point estimates varied only slightly (between 1.52 and 1.57).

## DISCUSSION

Our results demonstrate that CF carriers are at increased risk for pneumonia following a diagnosis of influenza. First, at an individual level, we found that the odds of developing pneumonia increased by approximately 34% in CF carriers following a diagnosis of influenza, compared with noncarriers diagnosed with influenza. Second, we found that while the incidence of influenza is not elevated among CF carriers, the incidence rate of pneumonia was significantly elevated among CF carriers compared with our control population across multiple influenza seasons. Given that approximately 3% of the United States population are CF carriers, our collective findings could have a substantial health impact at a population level and help explain why some people are more likely to acquire secondary pneumonia than others after an influenza infection.

CF carriers were not always thought to be at increased risk for pulmonary infections and other CF-related diseases [13, 14]. Historically, it was believed that approximately 50% of normal CFTR function was sufficient for maintaining health [21]. However, early studies of CF carriers were likely too small to detect increased levels of disease risk associated with the CF carrier state [22]. Larger and more recent studies demonstrate that CF carriers are indeed at increased risk of respiratory infections as well as almost all CF-related diseases [15, 23]. Despite the increased risk for pulmonary infections associated with the CF carrier state, it is substantially less than the risk associated with CF [15]. Furthermore, merely being a CF carrier may not be sufficient for developing recurrent pulmonary infections, and other genetic and or environmental exposures are likely needed for CF carriers to experience increased levels of CF-related conditions [22]. We think that influenza could be among the environmental exposures that increase the risk for pulmonary infections in CF carriers.

The link between influenza and bacterial pneumonia was described >100 years ago [12] and has been firmly established in clinical studies [24] and animal models and through mechanistic investigations [25]. Interestingly, prior influenza infections do not seem to increase the risk for all bacterial pneumonias. Instead, influenza specifically increases appears to increase the risk for pneumonia caused by *Streptococcus pneumoniae, S aureus,* and *Haemophilus influenzae* [24]. Multiple mechanisms are thought to play a role, with influenza increasing the risk for bacterial pneumonia (eg, decreased mucociliary clearance or virus-induced airway epithelial damage in the immune system) [11, 25].

Relevant to our findings for CF carriers, influenza infections particularly appear to inhibit CFTR activity [12, 26]. Reducing CFTR function in CF carriers who have only a 50% baseline level of CFTR function before an influenza infection could help explain why CF carriers are at increased risk for bacterial pneumonia. Multiple investigations indicate that influenza infections can adversely affect CFTR activity [12, 26, 27]. For example, the M2 protein of influenza A, the most common cause of influenza, both decreases CFTR activity and its expression [28]. The decrease in CFTR activity could increase risk for pneumonia through several mechanisms. First, decreased CFTR activity lowers ciliary beat frequency and mucociliary clearance, leading to reduced clearance of bacterial pathogens [29]. Second, CFTR plays an important role in controlling the pH of airway surface liquid fluid, enhancing the killing of bacteria by defensins (ie, antimicrobial peptides) in airway surface liquid [30]. Third, CFTR activity can affect inflammatory responses [31].

Our work has several limitations. First, we used only administrative data and did not have laboratory results, imaging, or clinical notes to validate the diagnostic codes we used. Second, many people do not seek care for influenza [17]; many cases presenting to healthcare visits are not diagnosed [32]; some patients might be more likely to be tested for influenza than others; and influenza testing practices likely changed during the study period. However, our second set of analysis specifically investigates the risk of pneumonia for CF carriers and does not rely on a prior diagnosis of influenza. Third, our data set included only people with private insurance or Medicare Advantage. Accordingly, our results may not be generalizable to people without private insurance, who may have different healthcare-seeking behavior.

Some additional limitations of our analysis resulted from how we identify CF carriers. For example, control subjects may not have received genetic screening, and we do not have access to the results of genetic panels that were performed in case patients. Thus, some of our control subjects may be misclassified, and some of our case patients might have undetected *CFTR* mutations not captured by standard screening panels. In addition, some CF carriers could have milder *CFTR* mutations, and our results might not be generalizable to all CF carriers. Finally, we identified CF carriers primarily through newborn screening or preconception testing; thus, our cohort is relatively young and primarily female. However, because older age is a risk factor for pneumonia [33], we may be underestimating the risk of pneumonia in CF carriers after influenza.

Despite these limitations, we found that CF carriers are at a substantially increased risk for pneumonia following influenza, identifying a new priority population for annual influenza vaccination. Furthermore, because 2%–11% of the population acquires influenza each year [34], and because there are >10–15 million CF carriers in the United States alone, a substantial number of pneumonia cases may be attributable to the CF carrier state. Although these results are exploratory, they highlight the need for future larger studies designed to estimate more precisely the risk for secondary influenza­–associated bacterial infections across the lifespan for CF carriers, especially at the extremes of age when the morbidity and mortality rates associated with from influenza are highest.

## Supplementary Material

ofaf642_Supplementary_Data

## Supplementary Data

Supplementary materials are available at *Open Forum Infectious Diseases* online. Consisting of data provided by the authors to benefit the reader, the posted materials are not copyedited and are the sole responsibility of the authors, so questions or comments should be addressed to the corresponding author.
