# Supplementary material for: The Increased Risk for Postinfluenza Pneumonia Among Cystic Fibrosis Carriers—A Population-Based Study
Source: Open Forum Infect Dis. 2025 Oct 23;12(11):ofaf642. doi: 10.1093/ofid/ofaf642 (PMC12575080; doi:10.1093/ofid/ofaf642)
Supplement: ofaf642_Supplementary_Data [file ofaf642_supplementary_data.docx]

**Appendix Material**

**Appendix Table 1 – ICD-9/10 diagnosis codes Used to identify influenza and pneumonia events**

| **Condition** | **ICD-9-CM Codes** | **ICD-10-CM Codes** |
| --- | --- | --- |
| Cystic Fibrosis | 2770, 27700, 27701, 27702, 27703, 27709 | E84, E840, E841, E8411, E8419, E848, E849 |
| Cystic Fibrosis Gene Carrier | V8381 | Z141 |
| Influenza | 487, 4870, 4871, 4878, 488, 4880, 48801, 48802, 48809, 4881, 48811, 48812, 48819, 4888, 48881, 48882, 48889 | J09, J09X, J09X1, J09X2, J09X3, J09X9, J10, J100, J1000, J1001, J1008, J101, J102, J108, J1081, J1082, J1083, J1089, J11, J110, J1100, J1108, J111, J112, J118, J1181, J1182, J1183, J1189 |
| Pneumonia | 00322, 0203, 0204, 0205, 0212, 0221, 0310, 0391, 0521, 0551, 0730, 0830, 1124, 1140, 1144, 1145, 11505, 11515, 11595, 1304, 1363, 4800, 4801, 4802, 4803, 4808, 4809, 481, 4820, 4821, 4822, 4823, 48230, 48231, 48232, 48239, 4824, 48240, 48241, 48242, 48249, 4828, 48281, 48282, 48283, 48284, 48289, 4829, 483, 4830, 4831, 4838, 4841, 4843, 4845, 4846, 4847, 4848, 485, 486, 5130, 5171 | A0103, A0222, A202, A212, A221, A310, A3701, A3711, A430, A481, B012, B052, B0681, B250, B371, B380, B381, B382, B390, B391, B392, B583, B59, B7781, J120, J121, J122, J123, J1281, J1289, J129, J13, J14, J150, J151, J1520, J15211, J15212, J1529, J153, J154, J155, J156, J157, J158, J159, J160, J168, J17, J180, J181, J188, J189, J851 |

**Appendix Table 2 – Baseline characteristics of influenza events included in the individual-level risk analysis**

|  | **CF Carriers**  N (%) | **Controls**  N (%) |
| --- | --- | --- |
| **Influenza Events** | 3820 (100) | 36247 (100) |
| **Secondary Pneumonia** | 116 (3.04) | 844 (2.33) |
| Concurrent influenza diagnosis | 54 (1.41) | 401 (1.11) |
| **Hospitalizations for Secondary Pneumonia** | 16 (0.42) | 129 (0.36) |
| Concurrent influenza diagnosis | 7 (0.18) | 51 (0.14) |
| **Age Group** |  |  |
| 0-4 | 376 (9.84) | 4352 (12.01) |
| 5-17 | 469 (12.28) | 4515 (12.46) |
| 18-29 | 1240 (32.46) | 10420 (28.75) |
| 30-39 | 1314 (34.4) | 13162 (36.31) |
| 40-49 | 341 (8.93) | 3227 (8.9) |
| 50-64 | 70 (1.83) | 528 (1.46) |
| ≥ 65 | 10 (0.26) | 43 (0.12) |
| **Female Sex** | 3197 (83.69) | 30100 (83.04) |
| **Month** |  |  |
| January | 808 (21.15) | 7876 (21.73) |
| February | 875 (22.91) | 8355 (23.05) |
| March | 569 (14.9) | 5155 (14.22) |
| April | 186 (4.87) | 1590 (4.39) |
| May | 71 (1.86) | 705 (1.94) |
| June | 44 (1.15) | 309 (0.85) |
| July | 21 (0.55) | 267 (0.74) |
| August | 37 (0.97) | 309 (0.85) |
| September | 70 (1.83) | 742 (2.05) |
| October | 180 (4.71) | 1702 (4.7) |
| November | 268 (7.02) | 2608 (7.2) |
| December | 691 (18.09) | 6629 (18.29) |
| **Year** |  |  |
| 2001 | 6 (0.16) | 23 (0.06) |
| 2002 | 8 (0.21) | 64 (0.18) |
| 2003 | 19 (0.5) | 177 (0.49) |
| 2004 | 3 (0.08) | 72 (0.2) |
| 2005 | 26 (0.68) | 243 (0.67) |
| 2006 | 21 (0.55) | 281 (0.78) |
| 2007 | 22 (0.58) | 294 (0.81) |
| 2008 | 103 (2.7) | 966 (2.67) |
| 2009 | 219 (5.73) | 2118 (5.84) |
| 2010 | 37 (0.97) | 413 (1.14) |
| 2011 | 88 (2.3) | 1112 (3.07) |
| 2012 | 88 (2.3) | 1124 (3.1) |
| 2013 | 156 (4.08) | 1707 (4.71) |
| 2014 | 284 (7.43) | 2707 (7.47) |
| 2015 | 171 (4.48) | 1580 (4.36) |
| 2016 | 188 (4.92) | 1710 (4.72) |
| 2017 | 344 (9.01) | 3440 (9.49) |
| 2018 | 506 (13.25) | 4724 (13.03) |
| 2019 | 549 (14.37) | 4821 (13.3) |
| 2020 | 504 (13.19) | 4255 (11.74) |
| 2021 | 84 (2.2) | 695 (1.92) |
| 2022 | 264 (6.91) | 2346 (6.47) |
| 2023 | 130 (3.4) | 1375 (3.79) |
| **Comorbidity Indicators** |  |  |
| Alcohol | 4 (0.1) | 59 (0.16) |
| Anemia | 36 (0.94) | 237 (0.65) |
| Arrhythmia | 74 (1.94) | 652 (1.8) |
| BloodLoss | 3 (0.08) | 28 (0.08) |
| CHF | 4 (0.1) | 55 (0.15) |
| Coagulopathy | 9 (0.24) | 66 (0.18) |
| Depression | 140 (3.66) | 1271 (3.51) |
| DM | 34 (0.89) | 351 (0.97) |
| DMcx | 17 (0.45) | 191 (0.53) |
| Drugs | 32 (0.84) | 247 (0.68) |
| FluidsLytes | 53 (1.39) | 573 (1.58) |
| HIV | 4 (0.1) | 20 (0.06) |
| HTN | 81 (2.12) | 949 (2.62) |
| Hypothyroid | 57 (1.49) | 438 (1.21) |
| Liver | 8 (0.21) | 105 (0.29) |
| Lymphoma | 1 (0.03) | 7 (0.02) |
| Mets | 0 (0) | 12 (0.03) |
| NeuroOther | 25 (0.65) | 234 (0.65) |
| Obesity | 70 (1.83) | 787 (2.17) |
| Paralysis | 1 (0.03) | 14 (0.04) |
| PHTN | 1 (0.03) | 35 (0.1) |
| Psychoses | 29 (0.76) | 263 (0.73) |
| PUD | 1 (0.03) | 10 (0.03) |
| Pulmonary | 236 (6.18) | 1996 (5.51) |
| PVD | 5 (0.13) | 29 (0.08) |
| Renal | 8 (0.21) | 58 (0.16) |
| Rheumatic | 13 (0.34) | 179 (0.49) |
| Tumor | 10 (0.26) | 77 (0.21) |
| Valvular | 6 (0.16) | 71 (0.2) |
| WeightLoss | 5 (0.13) | 45 (0.12) |

**Appendix Table 3 – Remaining regression estimates for study 1 (see Table 2 for primary effect estimates).**

|  | **Any Secondary Pneumonia** | **Hospitalization for Secondary Pneumonia** |
| --- | --- | --- |
| **Month** |  |  |
| January | Reference | Reference |
| February | 1.00 (0.82-1.23) | 1.18 (0.71-1.97) |
| March | 1.04 (0.82-1.31) | 1.12 (0.62-2.00) |
| April | 0.96 (0.67-1.37) | 0.99 (0.41-2.37) |
| May | 2.15 (1.47-3.14) | 2.49 (0.98-6.35) |
| June | 1.41 (0.76-2.59) | 2.26 (0.61-8.37) |
| July | 2.72 (1.60-4.59) | 1.15 (0.17-7.87) |
| August | 2.09 (1.24-3.53) | 0.81 (0.14-4.81) |
| September | 1.87 (1.26-2.78) | 1.34 (0.39-4.58) |
| October | 1.36 (0.97-1.90) | 1.75 (0.77-3.95) |
| November | 1.19 (0.88-1.61) | 0.69 (0.26-1.80) |
| December | 0.87 (0.68-1.11) | 0.63 (0.33-1.20) |
| **Year** |  |  |
| 2001 | Reference | Reference |
| 2002 | 0 (0-1.94e+139) | 1.02 (0-Inf) |
| 2003 | 1.19 (0.25-5.6) | 5871411.92 (0-Inf) |
| 2004 | 1.43 (0.28-7.44) | 4085094.55 (0-Inf) |
| 2005 | 0.88 (0.19-4.09) | 0.99 (0-Inf) |
| 2006 | 0.47 (0.1-2.31) | 2200727.31 (0-Inf) |
| 2007 | 0.54 (0.11-2.58) | 607814.71 (0-Inf) |
| 2008 | 0.49 (0.11-2.19) | 834480.54 (0-Inf) |
| 2009 | 0.34 (0.08-1.49) | 757505.99 (0-Inf) |
| 2010 | 0.75 (0.17-3.38) | 1580441.32 (0-Inf) |
| 2011 | 0.51 (0.12-2.26) | 444645.28 (0-Inf) |
| 2012 | 0.38 (0.08-1.67) | 585003.34 (0-Inf) |
| 2013 | 0.33 (0.07-1.44) | 1826870.01 (0-Inf) |
| 2014 | 0.22 (0.05-0.98) | 1076333.52 (0-Inf) |
| 2015 | 0.41 (0.09-1.81) | 1463890.29 (0-Inf) |
| 2016 | 0.4 (0.09-1.74) | 1093028.56 (0-Inf) |
| 2017 | 0.28 (0.06-1.22) | 936426.41 (0-Inf) |
| 2018 | 0.3 (0.07-1.3) | 555623.91 (0-Inf) |
| 2019 | 0.32 (0.08-1.39) | 841871.44 (0-Inf) |
| 2020 | 0.26 (0.06-1.12) | 629630.34 (0-Inf) |
| 2021 | 0.31 (0.07-1.42) | 876428.55 (0-Inf) |
| 2022 | 0.18 (0.04-0.78) | 93841.44 (0-Inf) |
| 2023 | 0.24 (0.05-1.07) | 142048.98 (0-Inf) |
| Comorbidity Indicators |  |  |
| Alcohol | 0.34 (0.04-2.67) | 0.00 (0.00-Inf) |
| Anemia | 1.45 (0.83-2.54) | 2.70 (1.07-6.86) |
| Arrhythmia | 1.61 (1.14-2.28) | 1.86 (0.95-3.64) |
| BloodLoss | 2.92 (0.83-10.33) | 4.34 (0.51-36.54) |
| CHF | 4.22 (2.02-8.82) | 13.55 (4.85-37.86) |
| Coagulopathy | 1.29 (0.44-3.79) | 4.57 (1.08-19.36) |
| Depression | 1.11 (0.80-1.55) | 2.83 (1.58-5.06) |
| DM | 0.82 (0.45-1.49) | 0.84 (0.27-2.60) |
| DMcx | 1.81 (1.00-3.29) | 1.50 (0.44-5.10) |
| Drugs | 2.03 (1.20-3.46) | 1.37 (0.43-4.35) |
| FluidsLytes | 2.65 (1.92-3.67) | 4.55 (2.54-8.13) |
| HIV | 2.36 (0.59-9.45) | 0.00 (0.00-Inf) |
| HTN | 1.15 (0.80-1.64) | 1.29 (0.63-2.64) |
| Hypothyroid | 1.49 (0.95-2.34) | 0.68 (0.18-2.60) |
| Liver | 1.85 (0.85-4.02) | 5.12 (1.57-16.68) |
| Lymphoma | 0.00 (0.00-Inf) | 0.00 (0.00-Inf) |
| Mets | 1.80 (0.21-15.63) | 0.00 (0.00-Inf) |
| NeuroOther | 2.55 (1.56-4.16) | 3.77 (1.60-8.88) |
| Obesity | 0.92 (0.60-1.41) | 1.37 (0.63-2.97) |
| Paralysis | 5.21 (1.32-20.59) | 23.72 (4.88-115.26) |
| PHTN | 1.02 (0.32-3.32) | 0.52 (0.09-2.96) |
| Psychoses | 1.49 (0.84-2.63) | 1.02 (0.29-3.64) |
| PUD | 1.27 (0.14-11.9) | 0.00 (0.00-Inf) |
| Pulmonary | 1.74 (1.39-2.18) | 2.56 (1.60-4.11) |
| PVD | 2.78 (0.90-8.63) | 11.55 (2.59-51.51) |
| Renal | 0.72 (0.24-2.14) | 0.86 (0.16-4.46) |
| Rheumatic | 1.40 (0.70-2.81) | 3.66 (1.32-10.15) |
| Tumor | 0.68 (0.20-2.37) | 0.86 (0.08-9.21) |
| Valvular | 1.82 (0.79-4.19) | 1.73 (0.40-7.45) |
| WeightLoss | 1.80 (0.61-5.33) | 1.94 (0.34-10.97) |

**Appendix Table 4 – Comparison of model performance and effect estimates with and without inclusion of an influenza interaction term: evaluating if there exists a influenza interaction effect with carrier status.** These results suggest that the effect of carrier status is additive rather than interactive with ILI activity. For both CDC reported ILI and an internally computed measure of ILI (using MarketScan data to compute weekly ILI incidence) the best performing model, in terms of AIC, was the model containing a single indicator for carrier status along with a term for 1-week lagged ILI activity (Models 1A and 1B). When an interaction term was added to the model (Models 2A and 2B), the model performance dropped and the interactive term conveyed an null effect; however, the primary additive effect of carrier status remained unchanged. When the lagged ILI term was removed from the model and replaced with the interaction term (Models 3A and 3B), model performance degraded even further. In this case, the primary additive effect appeared to diminish only slightly while the interaction term appears to capture some of the impact of removing the lagged ILI component. Finally, when only the interaction term was included (Models 4A and 4B), model performance degraded even further, while the interaction effect appears to capture part of the effect of both carrier status and lagged ILI activity. Together these results suggest that carrier status conveys an additive effect of uniformly increasing the risk of pneumonia relative to controls (conveyed in terms of odds ratios) rather than an interactive effect where the risk among carriers relative to controls varies with influenza activity.

| Using Exogenous (CDC Reported) ILI | | | | |
| --- | --- | --- | --- | --- |
|  | Model 1A | Model 2A | Model 3A | Model 4A |
| **CF Carrier** | 1.55  (1.50-1.60) | 1.59  (1.50-1.68) | 1.45  (1.37-1.53) |  |
| **CDC ILI** (1-wk lag) | 1.09  (1.08-1.10) | 1.09  (1.08-1.10) |  |  |
| **CF Carrer : CDC ILI** (1-wk lag) |  | 0.99  (0.97-1.01) | 1.03  (1.01-1.05) | 1.15  (1.13-1.16) |
| **AIC** | 11452.66 | 11453.23 | 11687.02 | 11845.86 |
| **BIC** | 11660.84 | 11667.19 | 11895.20 | 12048.25 |
| Using Internally (MarketScan) Computed ILI^1^ | | | | |
|  | Model 1B | Model 2B | Model 3B | Model 4B |
| **CF Carrier** | 1.54  (1.49-1.59) | 1.56  (1.50-1.62) | 1.50  (1.45-1.56) |  |
| **MarketScan Influenza Incidence** (1-wk lag) | 1.01  (1.01-1.01) | 1.01  (1.01-1.01) |  |  |
| **CF Carrer : MarketScan Influenza Incidence** (1-wk lag) |  | 1.00  (0.99-1.00) | 1.00  (1.00-1.01) | 1.02  (1.02-1.02) |
| **AIC** | 11550.14 | 11550.45 | 11686.56 | 12111.90 |
| **BIC** | 11758.33 | 11764.41 | 11894.74 | 12314.30 |

Note: All models also control for month and year of the observation period. ^1^ILI was computed using all influenza events recorded in a given week among patients enrolled in the MarketScan database. This was computed as the number of patients with an ILI-related visit during a given week, divided by the number of enrollees observable in that week.

**Appendix Table 5 – Sensitivity analysis of primary effect estimate and model performance to different lag terms in the exogenous ILI component.** The model with a single 1-week lag ILI component was deemed the best model for three reasons: (1) this was the most parsimonious model that was most theoretically consistent with the hypothesized relationship (i.e., around a 1 week lag between influenza and secondary infection), (2) this was the preferred model based on BIC values, (3) although the higher-order nested models were preferred by AIC, none of the nested ILI terms other than the 1-week lag were statistically significant.

| **Single ILI Component** | | | | | |
| --- | --- | --- | --- | --- | --- |
|  |  |  |  |  |  |
| **CF Carrier** | 1.55  (1.50-1.60) | 1.55  (1.50-1.60) | 1.55  (1.50-1.60) | 1.55  (1.50-1.60) | 1.55  (1.50-1.60) |
| **ILI Terms** |  |  |  |  |  |
| Concurrent | 1.08  (1.07-1.09) |  |  |  |  |
| 1-week lag |  | 1.09  (1.08-1.10) |  |  |  |
| 2-week lag |  |  | 1.08  (1.07-1.09) |  |  |
| 3-week lag |  |  |  | 1.07  (1.06-1.08) |  |
| 4-week lag |  |  |  |  | 1.05  (1.04-1.07) |
| **AIC** | 11471.70 | 11452.66 | 11490.24 | 11551.19 | 11593.18 |
| **BIC** | 11679.90 | 11660.84 | 11698.41 | 11759.34 | 11801.32 |
| **Nested ILI Terms** | | | | | |
|  |  |  |  |  |  |
| **CF Carrier** | 1.55  (1.50-1.60) | 1.55  (1.50-1.60) | 1.55  (1.50-1.60) | 1.55  (1.50-1.60) | 1.55  (1.50-1.60) |
| **ILI Terms** |  |  |  |  |  |
| Concurrent | 1.08  (1.07-1.09) | 1.02  (0.99-1.05) | 1.01  (0.98-1.05) | 1.01  (0.98-1.05) | 1.01  (0.98-1.05) |
| 1-week lag |  | 1.07  (1.04-1.10) | 1.09  (1.03-1.16) | 1.07  (1.01-1.14) | 1.07  (1.01-1.14) |
| 2-week lag |  |  | 0.98  (0.95-1.02) | 1.04  (0.97-1.10) | 1.02  (0.96-1.09) |
| 3-week lag |  |  |  | 0.96  (0.93-1.00) | 0.99  (0.93-1.06) |
| 4-week lag |  |  |  |  | 0.98  (0.94-1.01) |
| **AIC** | 11471.70 | 11453.53 | 11452.63 | 11447.40 | 11440.13 |
| **BIC** | 11679.90 | 11667.50 | 11672.37 | 11672.90 | 11671.40 |

**Appendix Table 6 – Sensitivity analysis of primary effect estimate to inclusion or exclusion of data prior to 2009 (i.e., low cohort size and sparse outcome measures) or after 2019 (i.e., when influenza incidence diminished during the COVID-19 pandemic)**

|  | **Incidence Rate Ratio** | **95% Confidence Interval** |
| --- | --- | --- |
| ***Years Included*** |  |  |
| 2001-2023 (Primary Results | 1.55 | 1.50, 1.60 |
| 2009-2023 | 1.57 | 1.52, 1.63 |
| 2009-2019 | 1.55 | 1.49, 1.61 |
| 2001-2019 | 1.52 | 1.46, 1.57 |

***Appendix Figure 1 – Number of enrollees represented across time in the CF carrier and control cohorts.*** *The y-axis depicts the total number of enrollee-months represented each month. Enrollee months were computed as the total days of enrollment in a given month summed across all enrollees observed in a given month, divided by the number of days in the month.*

***Appendix Figure 2 – Incidence of influenza and pneumonia by age for CF carriers (red) and controls (teal)***

******

***Appendix Figure 3 – Weekly incidence of pneumonia for CF carriers (red) and controls (teal).***

**
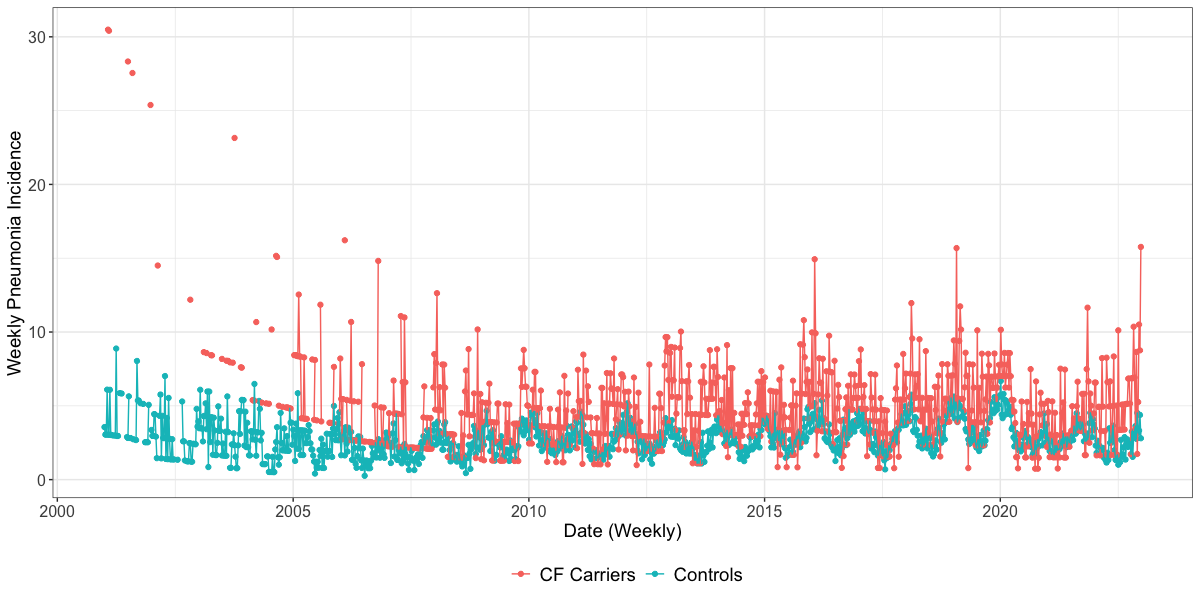
**
